# Type VI choledochal cyst: A case report

**DOI:** 10.1016/j.ijscr.2020.02.033

**Published:** 2020-02-19

**Authors:** Vicky S. Budipramana, Putu Ayu Saraswati

**Affiliations:** Digestive Surgery Division of Surgery Department, Dr. Soetomo Hospital Surabaya, Faculty of Medicine, Universitas Airlangga, Jl. Mayjen Prof. Dr. Moestopo 47, Surabaya, 60132, Indonesia

**Keywords:** Type VI choledochal cyst, Cystic duct, Case report

## Abstract

**Introduction:**

Choledochal cyst originating from the cystic duct is a very rare case and difficult to diagnose preoperatively, it is classified as Todani type VI choledochal cyst. Only three cases of type VI choledochal cyst have been reported in the literature.

**Presentation of case:**

A 35-year-old woman came with an enlarged and palpable mass in the right upper abdomen. The initial abdominal contrast CT scan and MRCP supported the presentation of Todani type 1 choledochal cyst. Intraoperatively, we found a different diagnosis, it was not type 1 but Todani type VI choledochal cyst. We found a large cyst originating from the cystic duct, attached to the gallbladder, without any connection to the intra or extrahepatic bile duct. The patient underwent simple cholecystectomy as well as excision of the cyst. After the follow-up for 3 months, the patient was uneventfully cured.

**Discussion:**

Preoperative diagnosis of type VI choledochal cyst seems to be difficult because it is a rare entity and it exhibits an ambiguous anatomic feature. Most cases may be misidentified as type I or II choledochal cyst before surgery. The diagnosis was made intraoperatively. Preoperatively the definitive anatomy may be delineated by ERCP if there is any high suspicion.

**Conclusion:**

Preoperative diagnosis of type VI choledochal cyst may be challenging and requires detailed radiological evaluation and multidisciplinary discussion. The cystic duct orifice and the diameter of the common bile duct are important to determine adequate management during the operation.

## Highlights

• Todani type VI choledochal cyst is a very rare case and difficult to diagnose preoperatively.
• Preoperative diagnosis of type VI choledochal cyst is difficult due to its rare entity and ambiguous anatomic feature.
• Preoperatively the definitive anatomy may be delineated by ERCP if there is any high suspicion.
• Preoperative diagnosis requires a detailed radiological evaluation and multidisciplinary discussion.

## 1 Introduction

Choledochal cyst is defined as congenital dilation of any portion of the bile duct, and the incidence reported was approximately 1: 100.000 to 1: 150.000 individuals in Western countries, and 1 in 13: 000 in certain parts of Asia [1]. Choledochal cyst can be found in the person of any age, but in contrast to the experience in pediatric patients, adult patients have an increased rate of associated hepatobiliary pathology. Similarly, female: male ratio has been found in adults [2]. The classification has changed from 3 types, described by Alonso-Lej, to 5 types, described by Todani, who combined Alonso-Lej’s classification and variants of Caroli disease. However, these classifications do not include isolated dilation of the cystic duct without common bile duct (CBD) or common hepatic duct (CHD) involvement [1,3]. Until recently, only three cases of type VI had been reported [1,2,4]. We present a rare case of type VI choledochal cyst and review to the literature in order to determine the basis of diagnosis and optimal management. This work has been reported in line with the SCARE criteria [5].

## 2 Presentation of the case

**Fig. 1:**
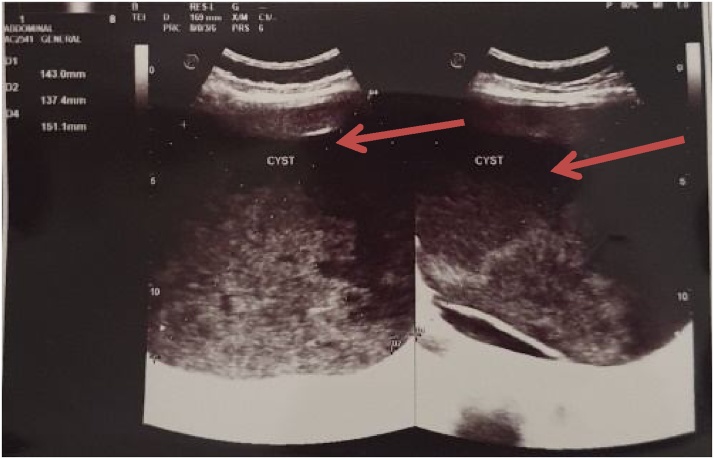
Pancreatic head and liver cyst were revealed during abdominal ultrasonography.

**Fig. 2:**
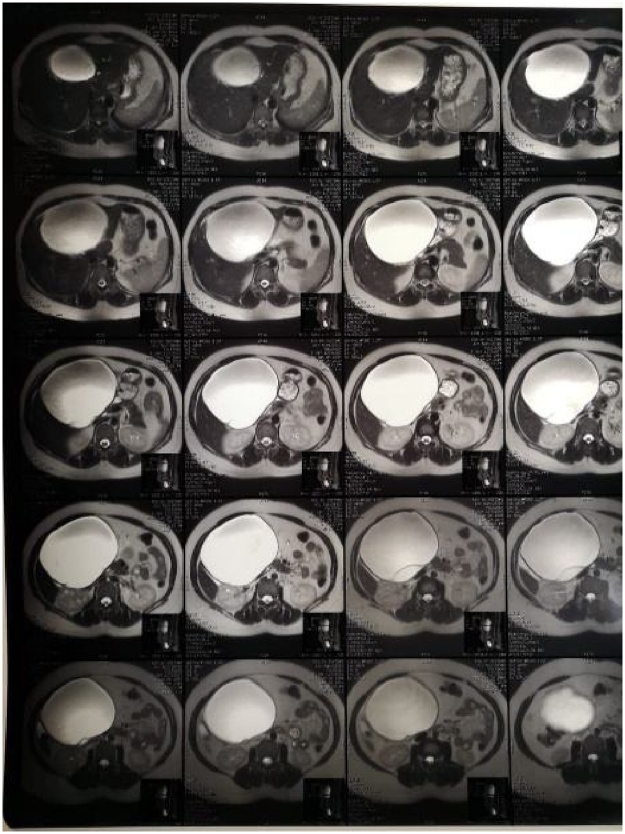
CBD dilatation with a cystic component (14HU) inside.

A 35-year-old woman came to the hospital with chief complaint about the enlarged mass in the right upper abdomen accompanied by a history of pain for the last 2 years and was getting worse for the last 1 month. She also complained about feeling nauseous and vomiting. Other symptoms including fever and jaundice did not occur. From the physical examination of the abdomen, we found a palpable mass in the right upper abdomen, sized 20 cm in diameter, fixed to the surrounding structure and no sign of tenderness. There were no abnormal findings on the laboratory test. The abdominal ultrasonography showed a pancreatic head separated with liver cyst (Fig. 1). Abdominal CT scan with contrast revealed CBD dilatation with cystic component (14HU) inside, which revealed pressing against the right side of gallbladder, pancreas and the left side of duodenum (Fig. 2), which supported the presentation of type 1 choledochal cyst according to Todani classification. Additional investigation with MRCP was made and resulted in the same conclusion.

**Fig. 3:**
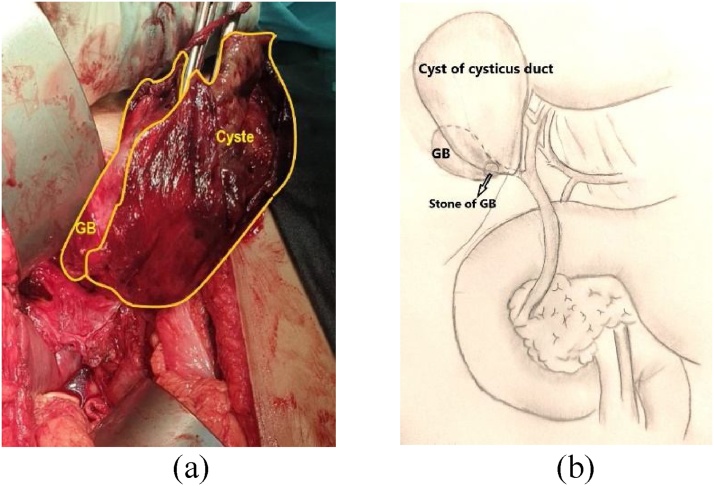
(a) A large fusiform cyst attached to the gallbladder; (b) A diagrammatic sketch to illustrate the cyst of cystic duct relationship to the gall bladder and common bile duct.

During the operation, we found different choledochal cyst from those described by Todani classification. We found a large cystic fusiform sized 14 × 14 × 1 cm attached to the gallbladder, the edge of the cyst attached to segment 3, 4, and 5 of the liver (Fig. 3). The gallbladder was contracted and a stone sized 2 cm was found. There is no connection between the cystic duct with CHD or CBD. When the cyst was aspirated, 1000 cc bile fluid was taken out. The cyst wall was thin and there was no sign of calcification. We performed a simple cholecystectomy as well as excision of the cyst.

**Fig. 4:**
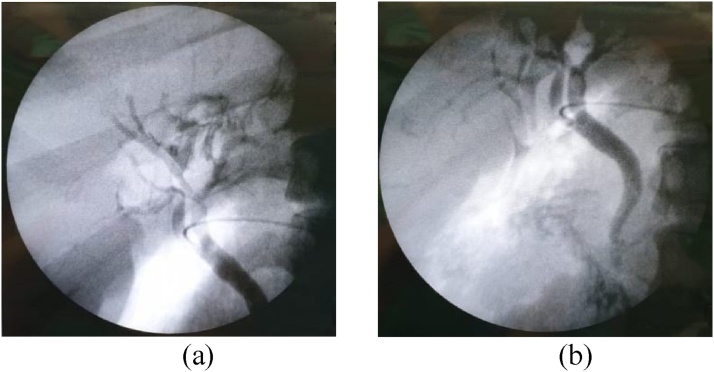
Intraoperative cholangiography performed during operation after cyst resection.

After we performed simple cholecystectomy and excision of the cyst, we continued with intraoperative cholangiography and there was no dilatation of intrahepatic bile duct and extrahepatic bile duct (Fig. 4). The specimens measured 14 cm in length, while GB was 9.2 cm and 0.5 cm in diameter. The patient was discharged without any specific postoperative morbidities or complications on the 6^th^ day.

**Fig. 5:**
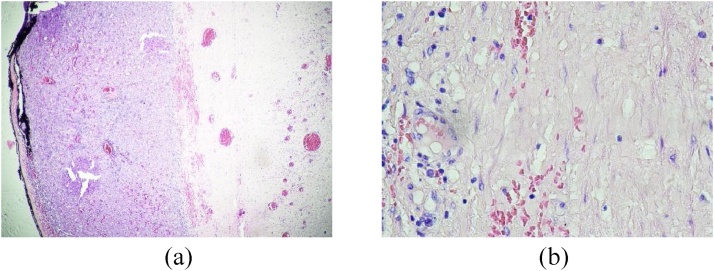
Pathology investigation of the cyst: (a) Thickened fibrous wall of the cyst; (b) Infiltration of polymorphonuclear and mononuclear on the cyst wall.

Pathology reports revealed that the cyst duct was dilated with thickened fibrous wall infiltrated by polymorphonuclear and mononuclear leukocytes (Fig. 5). The edge of the cyst consisted of proliferation of hepatocyte cells. There was chronic inflammation in the wall of the GB but no specific process and sign of malignancy. At the postoperative follow-up on the 3^rd^ month, the patient had completely recovered but future annual follow-up will be necessary.

## 3 Discussion

Cystic malformation of the cystic duct was first suggested as another type of biliary cyst by Serena Serradel et al. It has been reported as isolated cystic duct dilation or cystic duct dilation associated with other findings, including fusiform dilation of CBD [2]. The clinical presentation of type VI choledochal cyst is like the other variant of choledochal cyst. Most cases are asymptomatic or may present with no specific upper abdominal pain located in epigastric area or right upper abdomen; obstructive jaundice or acute cholangitis develops in some cases. In present case few cases, abdominal pain and vomiting are the only related findings [1,6]. Jaundice may be present due to mass effect to the cyst to CBD [3,7]. An accurate preoperative diagnosis of type VI choledochal cyst seems to be difficult because it is a rare entity and it exhibits ambiguous anatomic feature. Most cases are misidentified as type I or II choledochal cyst before the operation. This condition might occur when fusiform dilation of CBD is also present as a type I choledochal cyst [2,6]. We re-examined the MRCP and the conclusion from radiologist is still fusiform dilation of the CBD. Some of the cases of type VI choledochal cyst were reported that the diagnosis was made also intraoperatively. The anatomy can be shown by ERCP preoperatively if MRI is not conclusive. Intraoperative cholangiogram is a useful adjunct on the table to define the biliary anatomy [8].

Multiple theories have been proposed to explain the origin of bile duct cyst. The most widely accepted hypothesis is Babbitt's theory which stated that cystic dilation of the bile ducts is related to an anomalous arrangement of the pancreaticobiliary ductal junction. The pancreaticobiliary junction is proximal to the sphincter of Oddi. An anomalous pancreaticobiliary junction is often associated with long common channel that predisposes to reflux of pancreatic juice into the biliary tree; leading to inflammation, ectasia, and finally dilation [3,6,7]. Most of the reported cases have normal intra and extrahepatic biliary ductal system, which indicates distinct pathology. The reason for isolated involvement of the cystic duct excluding the CBD is unclear; possibly, the junction of the cystic duct with CBD is the weakest part due to the least vascularity, causing an ecstatic change that continues as a vicious cycle, resulting in further dilatation [4].

The appropriate management of type VI choledochal cyst is guided by the morphology, based on the cystic duct opening to CBD. The surgery recommended for the type VI choledochal cyst with narrow-based cystic duct or normal cystic duct between the cyst and CBD is simple cholecystectomy with cyst excision neither open nor laparoscopic [1,4,6,9]. Cystic duct cysts with wide openings to the CBD are usually accompanied by an abnormal CBD, so excision of the cyst en bloc with the gallbladder and distal CBD then continued with bilioenteric reconstruction [1,9]. Malignant neoplasms of the biliary tract develop in 10%–15% of patients with choledochal cyst and the risk of malignancy increases with age [2,7,9]. Therefore, the development of malignancy in type VI choledochal cyst is a rare presentation. Proved malignancy of the cyst with CBD or gallbladder requires definitive procedure in the form of Whipple’s operation or radical cholecystectomy [1,9].

## 4 Conclusion

It is difficult to identify type VI choledochal cyst preoperatively. Preoperative diagnosis may be challenging and requires a detailed radiological evaluation and multidisciplinary discussion between the surgeon and radiologist. Knowledge of this anatomical variant is important to every hepatobiliary surgeon. The cystic duct orifice and the diameter of the CBD are important for determining adequate management during the operation. The surgical management of such cases ranges from simple cholecystectomy to biliary resection procedures.

## Funding

This research did not receive any specific grant from funding agencies in the public, commercial, or not-for-profit sectors.

## Ethical approval

Our case is exempt from ethical approval in our institute.

## Consent

Written informed consent was obtained from the patient for publication of this case report and accompanying images. A copy of the written consent is available for review by the Editor-in-Chief of this journal on request.

## Author contribution

**Vicky S. Budipramana:** study concept and design, paper writing and editing.

**Putu Ayu Saraswati:** data collection.

## Registration of research studies

This manuscript is a case report, not a research study.

## Guarantor

The guarantor of this study is Vicky S. Budipramana.

## Provenance and peer review

Not commissioned, externally peer-reviewed.

## Declaration of Competing Interest

We don’t have any conflicts of interest with any person or organization.
